# Supplementary material for: Diagnostic accuracy of a SARS-CoV-2 rapid antigen test among military and civilian personnel of an Air Force airport in central Italy
Source: PLoS One. 2022 Nov 28;17(11):e0277904. doi: 10.1371/journal.pone.0277904 (PMC9704652; doi:10.1371/journal.pone.0277904)
Supplement: Attachment — Submitted filename: Response to Reviewers.docx [file pone.0277904.s003.docx]

**Point-by-point response to Reviewers**

We thank Reviewer #2 for her/his further requests: they allowed us to improve further the manuscript.

**Reviewer #2**

Reviewer comment 1:

1. The authors should elaborate more about the background of the study by copy and paste your response ‘The study was part of the public health response to control as soon as possible any outbreak occurring in the military airport (as reported in the Scientific Collaboration Protocol signed by the Experimental Flight Center, Italian Air Force Logistic Command and Istituto Superiore di Sanità on 30 November 2020), with simultaneous evaluation of the RADT used for the screening.’ into line 95, after the sentence ‘………….transmission of SARS-CoV-2 infection in the workplace.’

Response:

The sentence was copy-pasted in the manuscript (line 95, Methods - Study population section) as suggested by the reviewer (line 100 in the revised version)

Reviewer comment 2:

2. Reviewer 1 and I shared similar concerns of using fluorescence based RADT in your study, it is worthwhile to justify the reasons for selecting this RADT instead of traditional RADT without using fluorescence instrument.

Response:

Although the test requires a fluorescence reader, the running time from sample to result is comparable to traditional RADTs; however, it provides the added value that the result is not subject to the operator's interpretation. In fact, the positive/negative output of the fluorescence reader is automatic, based on an algorithm comparing the fluorescence obtained from the sample to a cut-off value; in the traditional RADTs, the operator can see more or less well a faint colored band of positivity

We included this explanation in the “Introduction” section of the manuscript (line 84, after “… in human NPSs within 20 minutes.”)

Reviewer comment 3:

3. Both reviewers 1 and 3 raised the concerns of similar studies have been published between 2020 and 2022. I also shared this view when I reviewed the first version, however, the PLOS journal did not focus on the novelty of the research. As I am not the new reviewer in PLOS, that’s why I did not raise this out. I suggest you should go through your manuscript thoroughly, try to write more about the background and the reasons to launch this study. The above comments 1 and 2 are aimed at filling this gap.

Response:

The manuscript was revised and modified in the “Introduction” and “Methods” section, according to the reviewer comments 1 and 2; in particular, we stressed that:

- the use of a fluorescence reader makes results less dependent from the operator interpretation than traditional tests

- the study population is unselected and mostly asymptomatic

- the study was carried out in the frame of a public health response, with simultaneous evaluation of the RADT used for screening
